# Epidemiology of Imported Malaria in Netrokona District of Bangladesh 2013-2018: Analysis of Surveillance Data

**DOI:** 10.1155/2019/6780258

**Published:** 2019-06-13

**Authors:** Md Abdul Karim, M. Moktadir Kabir, Md Ashraf Siddiqui, Md Shahidul Islam Laskar, Anjan Saha, Shamsun Naher

**Affiliations:** ^1^Communicable Disease (Malaria) Programme, BRAC, Bangladesh; ^2^Communicable Disease (Malaria) and Water Sanitation & Hygiene Programme, BRAC, Bangladesh; ^3^National Malaria Elimination Programme, Bangladesh

## Abstract

**Introduction:**

Netrokona is one of the first phase malaria elimination targeted 8 districts of Bangladesh by 2021. The district constitutes only 7% of the population but contributes half of the malaria cases in that area. Most of the cases of that district are imported from Meghalaya State of India. The study was conducted to understand the epidemiology of these imported malaria cases for further strategy development to prevent both imported and introduced cases.

**Methodology:**

The study was retrospectively conducted on the malaria cases confirmed by microscopy and/or RDT by the government and/or NGO service providers between 2013 and 2018. The information of the cases was collected from the verbal “investigation” report of individual malaria confirmed cases. The respondents of the “investigation” were either the patients or their family members. Out of the 713 cases during the study period, descriptive analysis of 626 cases (based on the completeness of “investigation form”) of the district was done using MS Excel version 2016.

**Results:**

Proportion of imported malaria in Netrokona district increased from 60% in 2013 to 95% in 2018 which persists throughout the year with a little seasonal fluctuation. The overall contribution of these imported cases is 93% by cross-border workers by population type and 84%, 66%, and 95% by male, labour, and tribal population considering the factors of sex, occupation, and ethnicity, respectively. Population aged between 15 and 49 years contributed 82% of these imported cases. All of these cases occurred in the internationally bordering belt with Meghalaya State of India. Species-wise distribution revealed lower* P. falciparum (63*%) and higher* mixed (28*%) infection in imported cases compared to the 71% Pf and 20% mixed infection among the indigenous infections whereas* P. vivax* is similar in both cases.

**Conclusion:**

Imported malaria is an emerging issue that has a potential risk of increased local transmission which might be a challenge to malaria elimination in that area. Appropriate interventions targeting the cross-border workers are essential to prevent the introduced cases and subsequently avoid reestablishment when elimination of the disease is achieved.

## 1. Introduction

**Figure 1:**
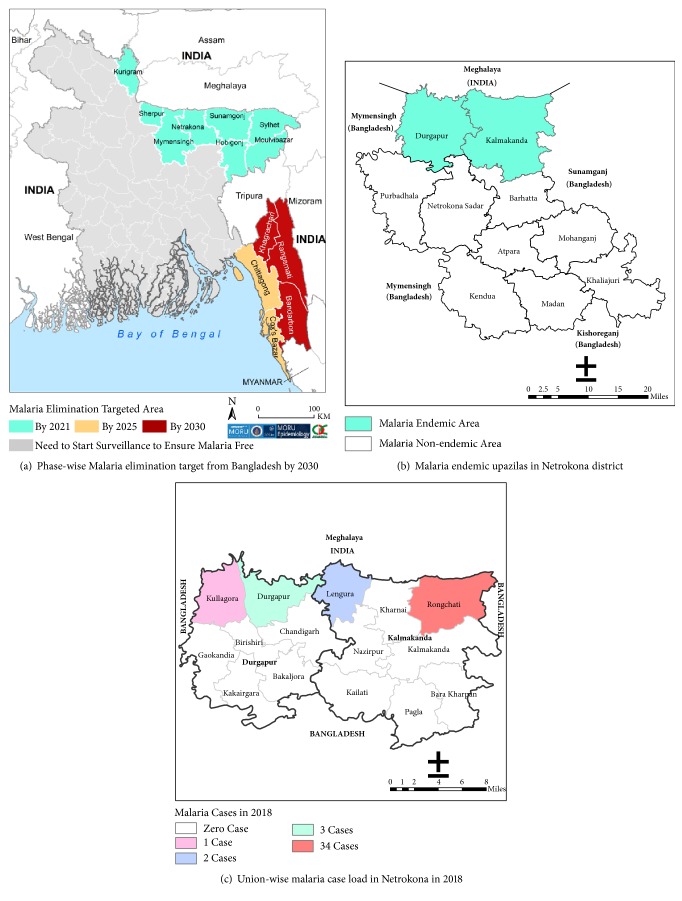


Bangladesh is one of the 87 malaria endemic countries and territories of the world in 2017 [1]. Currently, a total population of 17.52 million in 13 bordering districts of the country are at risk of the disease [2]. With the financial support from the Global Fund, the country intensified the programme activities in partnership between government and BRAC led NGO consortium and achieved remarkable reduction of malaria burden in the last ten years. This results in shifting the country strategy from control to elimination of the disease through three phases: 2021, 2025, and 2030 [2] (Figure 1(a)). The north and northeast 8 districts of the country are the first phase malaria elimination targeted areas of the country by 2021 [2].

Netrokona is one of these first phased malaria elimination targeted districts of the country. The district is situated in the northern part of Bangladesh. Out of the total 10 administrative upazilas (subdistrict), 2 upazilas (consisting of 15 unions) with internationally bordering with Meghalaya State of India are historically malaria endemic (Figure 1(b)). The endemic upazilas of the district constitute 7% of the malaria endemic population of the north and northeast 8 districts of the country whereas they contribute half of the malaria burden of that area [2, 3]. The majority (>60%) of the population, especially in the international border-belt of the endemic area (Figure 1(c)), is tribal aborigines contributing the highest number of malaria cases of the district [2]. Preelimination activities were initiated and surveillance system was strengthened in the district since 2012 and subsequently elimination was started in 2017.

The surveillance data show that most of the cases of the district are imported from Meghalaya State of India [4]. This imported malaria has a potential risk of increased local transmission and might cause the reintroduction of local cases, which could be a major challenge for elimination of the disease from that area [5–7]. This challenge warrants the conducting of the study to understand the epidemiology of the imported cases for future strategy development required to prevent both imported and introduced malaria with appropriate interventions.

## 2. Methodology

### 2.1. Surveillance and Case Investigation

Like other first phased malaria elimination targeted districts, active and passive surveillance are in place in Netrokona. Service providers are available from facilities down to the community in the whole endemic area. The catchment area is fixed for every service provider who frequently moves and searches for malaria cases by conducting household visits, organising fixed and mobile health camps at the community. When any malaria case is diagnosed, surveillance by community health workers, such as searching for malaria suspects within households within a 500-metre radius of the index case or the nearest 60 households, whichever is less, performing parasitological tests for malaria within 3 days, and follow-up of this surroundings for next 30 days, is done to identify if any reactive case is transmitted from the index case. Besides, the service providers have the list of cross-border workers (which varies between 700 and 800; list is updated accordingly) who work in the Meghalaya State of India and contribute most of the imported cases of the district. The parasitological tests are done at the time of their returning to home from their work. Subsequently, they are followed up to ensure the diagnosis and treatment services if their malaria symptoms appear later on. Thus, the strengthened surveillance system covered the diagnosis and treatment services of all the malaria cases (there were no missing cases) of the district during the period.

When one malaria case is confirmed by Rapid Diagnostic Test (RDT) and/or microscopy, detailed information of it is sent to national programme and NGO central level through mobile SMS immediately. The detailed investigation of the case is done verbally by local management staff (government and/or NGO) using the prescribed form of the programme. The respondents of the investigation are either the cases or their family members. After completion of the investigation, the investigators classify the source of infection based on the traveling, outstay, and dwelling history of the case during last one month, considering the incubation period of the disease.

### 2.2. Study Design, Data Collection, and Analysis

The study was retrospectively conducted on the malaria cases confirmed by Rapid Diagnostic Test (RDT) and/or microscopy by the government and/or NGO service providers in the study site between 2013 and 2018. A total of 713 malaria cases (601 cases by NGO service providers and 112 cases by government service providers) were diagnosed. Out of which, 626 cases were investigated. The source of infection of 87 cases (in 2013: 55; in 2014: 26; in 2015: 1; and in 2017: 5) remained unknown, so they were excluded from the analysis.

The completed “investigation” reports of individual cases were collected and preserved in the central level. These data of “investigation” report was entered into MS Excel version 2016 and the descriptive analysis was done for age, sex, occupation, species, and seasonal and geographical distribution of the cases. The comparison of these factors between imported and indigenous infections has been done tabularly and/or graphically and presented in the report.

### 2.3. Operational Definition

In this study,* “imported malaria” has been defined as “the malaria case occurring outside the national boundary but diagnosed within the national boundary” which is measured by the individual's history of staying in malaria endemic country within previous one month, considering the incubation period of the disease*.* “Cross-border workers”* refer to the inhabitants (Bangladeshi citizens) of the endemic area of Netrokona district who cross the international border between Bangladesh and India for their occupational purpose, stay in Meghalaya State of India, and work there. The two malaria endemic upazilas represent district and will be referred to as* “Netrokona district”*.* “Static population”* refers to the inhabitants of endemic area of the district who do not stay at night outside the international border for occupation purpose like cross-border workers.

### 2.4. Ethical Consideration

The ethical issues such as anonymity of the respondents were maintained, and nothing was done during the study which could hamper the regular activity of the respondents or programme or be harmful for the cases, respondents, or other family members.

## 3. Results

**Figure 2:**
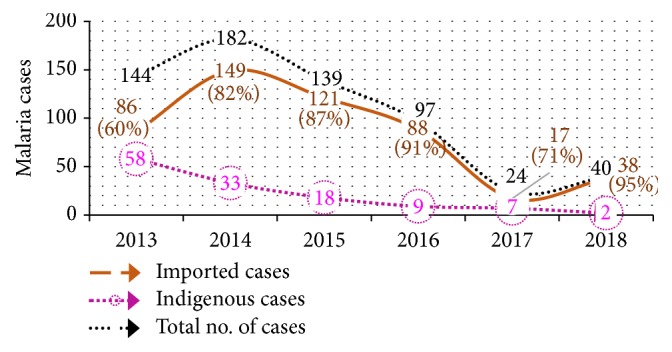
Trend of malaria cases in Netrokona district: 2013-2018.

**Figure 3:**
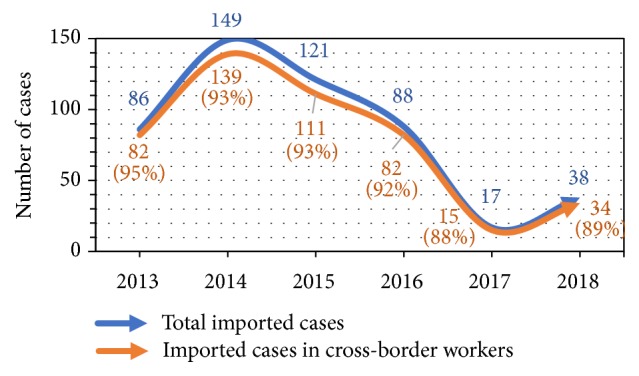
Trend of imported cases and contribution by cross-border workers.

Between 2013 and 2018, imported cases contributed 60% to the 95% malaria burden in Netrokona district (Figure 2). The mean age of these imported cases is 32 years and most of the cases (82%) were between 15 and 49 years old. The distribution of these imported cases by population type showed overall 93% (88% to 95%) contribution by the cross-border workers (Figure 3).

Distribution of the malaria cases by different factors such as sex, occupation, and ethnicity revealed the contribution of the imported cases by male (84%), labour (66%), and tribal population (95%) which is quite higher in each category compared to the same one for indigenous infection.

**Figure 4:**
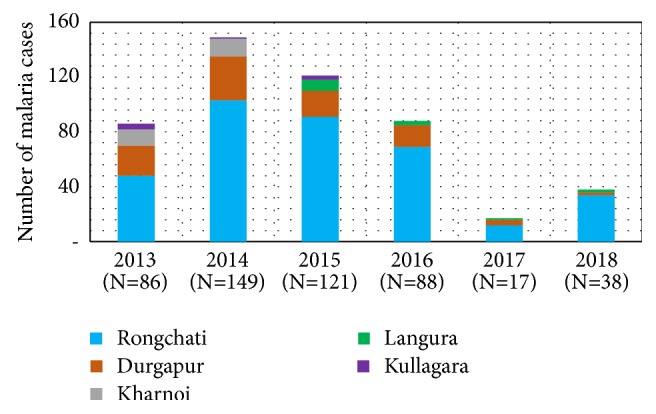
Union-wise distribution of imported cases: 2013-2018.

**Figure 5:**
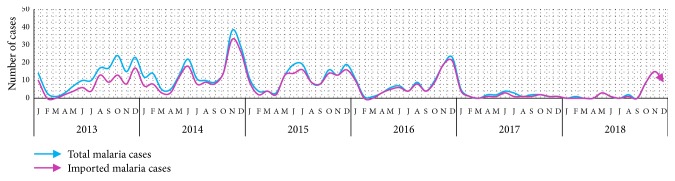
Seasonal trend of total versus imported malaria cases in Netrokona district: 2013-2018.

Geographical distribution revealed that all the imported cases occurred in populations of bordered (with Meghalaya, India) 5 unions constituting 28% of the endemic population of the district (Figure 4). Imported cases occurred throughout the year with seasonal fluctuation (Figure 5). Species-wise distribution shows lower* P. falciparum (63*%) and higher* mixed (28*%) infection among imported cases compared to the indigenous (71% Pf and 20% mixed) infection whereas* P. vivax *is similar in cases of both types (Table 1).

## 4. Discussion

**Figure 6:**
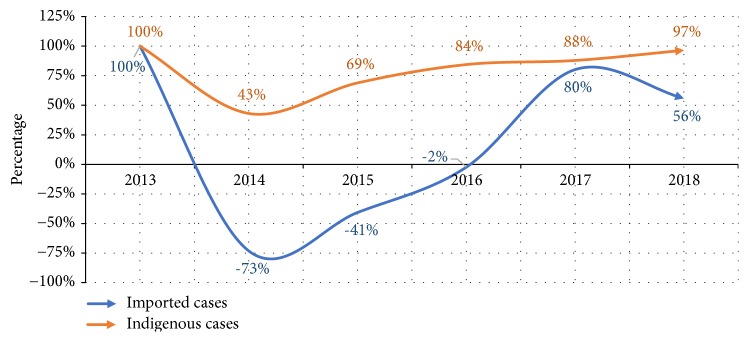
Reduction of imported and indigenous malaria infection in Netrokona compared to the year 2013.

Imported malaria is an emerging issue in the elimination settings in many countries [8, 9]. This study reviewed the situation of imported malaria in Netrokona district of Bangladesh where malaria burden has reduced by 91% between 2008 and 2018 and elimination of the disease by 2021 is the goal [2, 10]. The higher reduction of indigenous (97%) infection, compared to imported (56%) cases between 2013 and 2018 (Figure 6), results in the increased portion of imported malaria burden of the district over the years. Like other elimination settings, imported malaria in this district is mostly contributed by adult male and labour by occupation [11].

**Figure 7:**
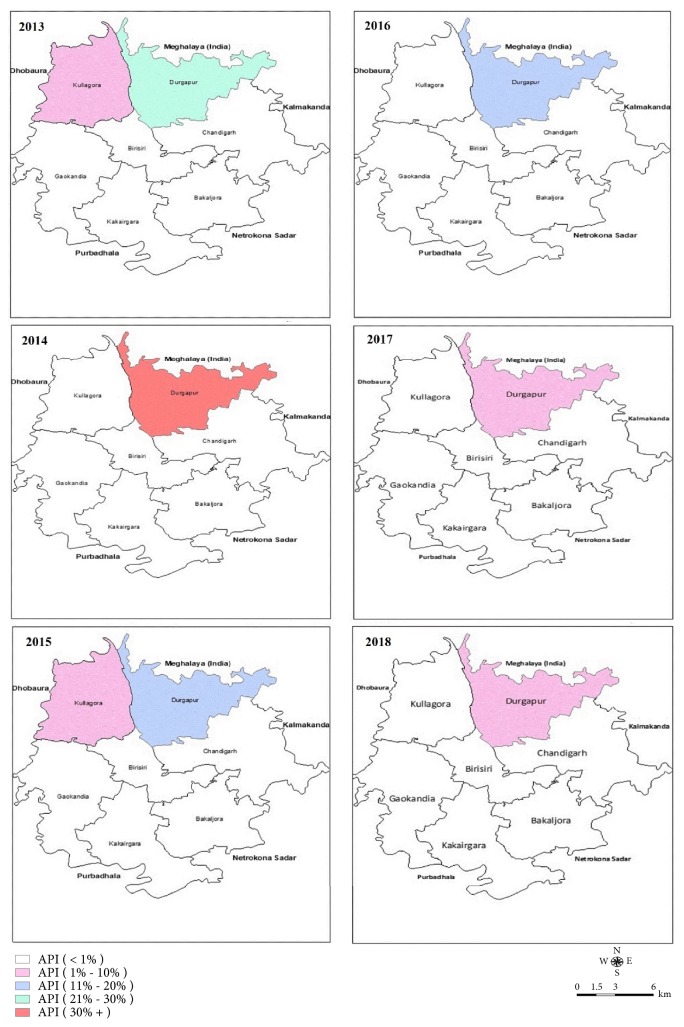
Union-wise Annual Parasite Incidence (API) in cross-border workers in Durgapur upazila: 2013-2018.

**Figure 8:**
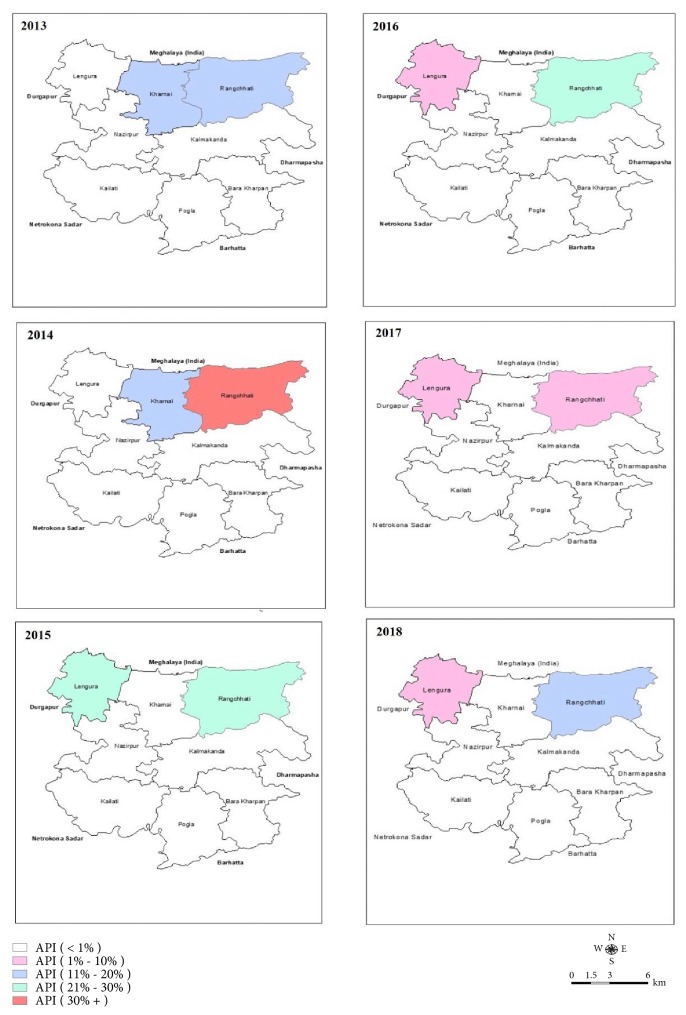
Union-wise Annual Parasite Incidence (API) in cross-border workers in Kalmakanda upazila: 2013-2018.

Due to geographic location of the area nearby the international border (between Bangladesh and India), the population have a very little scope of work within the national boundary. Based on the accessibility and scope of work in the bordering area of Meghalaya, the number of cross-border workers and their contribution to the imported cases differ in the bordering unions of the district (Table 2, Figures 7 and 8). Therefore, breadwinners including other family members cross the border and work in high malaria endemic Meghalaya State of India throughout the year [12]. Males mostly work as coalmine-labour in the winter and wood-cutter in the rainy season on a contract basis. They took their adolescent boys there to support their contracted work. They need to stay there continuously almost for one month for their work. They become higher susceptible to mosquito bites due to outdoor work, living in poor conditioned houses, lack of personal protection, immunity, and awareness and bear the most of the imported malaria cases [9, 13]. Adult female members are involved in other activities such as household work or running a shop and supply of food to the labours. Children also need to cross the border when their parents went there for long period. They (cross-border workers and their family members) stay there without personal protection from mosquito bites as many of them do not hang Long Lasting Insecticidal Mosquito Nets (LLINs) regularly before sleeping at night due to their fatigue after laborious work from dawn to dusk. Thus, these population groups become infected and contribute to the increased imported malaria cases compared to indigenous cases. Larger number of imported* P. vivax *and mixed (Pf and Pv) species infection in imported cases can be one of the major challenges to malaria elimination due to dormant hypnozoites in liver cells and parasite transmission from gametocytes before appearance of symptoms [12, 14]. In addition, people with different occupations such as farmers, students, housewives, service holders, teachers, and businessmen, living in the bordering villages need to work in hilly Meghalaya border and frequently cross the border (but do not stay there at night) due to the location of their lands and houses, and they contribute to the imported cases. Besides, some Indian citizens living in bordering area receive malaria diagnosis and treatment services in Bangladesh through their relatives and sometimes they stay in Bangladesh (in their relatives' houses) during treatment. Thus movement of population between endemic/high endemic and nonendemic/low endemic countries due to globalization/occupational purposes has increased the emergence of imported malaria [9, 11]. Consequently, cross-border workers can contribute a significant number of imported cases, which has the potential risk of local transmission of the disease to the area where the transmission has already been interrupted and, thus, can be a major challenge to malaria elimination [6, 7, 11]. It is an increasing problem in many countries for the last decades and caused thousands of cases worldwide and large number of deaths every year [9–11, 13]. History of malaria outbreaks due to introduced cases in the elimination settings evidences the imported cases as challenge to malaria elimination [15–18].

In-depth orientation of cross-border workers on malaria; standby treatment during staying in high endemic zone; continuation of parasitological tests of all individuals during entrance to the country from Meghalaya; continuation of follow-up of the returnee (from coalmine and/or forest); universal access of cross-border worker by distributing supplementary LLINs; and ensuring their utilization during staying in endemic zone can be pivotal to prevent introduced malaria and reestablishment of the disease when elimination will be achieved.

## 5. Limitations of the Study

Some cases diagnosed by the government service providers were left out of the investigation especially during the first two years of the initiation of the preelimination activity. These cases were excluded from the analysis. Due to verbal investigation of the cases, potential of recall bias of the respondents cannot be avoided. The results could not be compared with the findings of other studies due to nonavailability of studies in similar settings, and the results might be unique in the district where the study was conducted.

## 6. Conclusion

Drastic reduction of indigenous malaria between 2013 and 2018 leads to increased portion of imported cases in Netrokona district. These imported cases constitute an emerging issue and have the potential risk of increasing local transmission which might be a challenge to malaria elimination in that area. Appropriate interventions targeting the high-risk population group (cross-border workers and their family members) are essential to prevent the introduced cases and subsequently avoid reestablishment when elimination of the disease is achieved.

## Figures and Tables

**Table 1:** Comparison between imported and indigenous cases by different factors.

Factors	Category	Imported cases		Indigenous cases	
		Number	%	Number	%
Age group (Year)	< 5	5	1%	3	2%
	5-14	32	6%	36	28%
	*15-49*	*408*	*82*%	*67*	*53*%
	50+	54	11%	21	17%

Population type	*Cross-border worker*	*465*	*93*%	*8*	*6*%
	Static population	17	3%	119	94%
	Indian citizen	17	3%	-	-

Sex	*Male*	*418*	*84*%	*86*	*68*%
	Female	81	16%	41	32%

Occupation	*Labour*	*328*	*66*%	*17*	*13*%
	Farmer	75	15%	31	24%
	Housewife	35	7%	27	21%
	Student	35	7%	31	24%
	Others	26	5%	21	17%

Ethnicity	*Tribal*	*472*	*95*%	*76*	*60*%
	Bengali	27	5%	50	40%

Species	P. falciparum (Pf)	*312*	*63*%	*91*	*71*%
	P. vivax (Pv)	46	9%	11	9%
	Mixed	*141*	*28*%	*25*	*20*%

**Table 2:** Union-wise present number of cross-border workers.

Name of Upazila	Name of Union	No. of cross-border workers
Durgapur	Durgapur	100
	Kullagara	238

Kalmakanda	Rongchati	317
	Lengura	34
	Kharnoi	67

*Total*		*756*

## Data Availability

The data used to support the findings of this study are available from the corresponding author upon request.
